# Oral Administration of α-Asarone Promotes Functional Recovery in Rats With Spinal Cord Injury

**DOI:** 10.3389/fphar.2018.00445

**Published:** 2018-05-07

**Authors:** Min-Jae Jo, Hemant Kumar, Hari P. Joshi, Hyemin Choi, Wan-Kyu Ko, J. M. Kim, Sean S. S. Hwang, Song Y. Park, Seil Sohn, Alvin B. Bello, Kyoung-Tae Kim, Soo-Hong Lee, Xiang Zeng, Inbo Han

**Affiliations:** ^1^Department of Neurosurgery, CHA Bundang Medical Center, CHA University, Seongnam-si, South Korea; ^2^Department of Biomedical Science, CHA University, Seongnam-si, South Korea; ^3^Department of Neurosurgery, School of Medicine, Kyungpook National University, Kyungpook National University Hospital, Daegu, South Korea; ^4^Department of Histology and Embryology, Zhongshan School of Medicine, Sun Yat-sen University, Guangzhou, China

**Keywords:** spinal cord injury, α-asarone, neuroprotection, anti-inflammation, M2 polarization

## Abstract

α-asarone, a bioactive compound found in Acorus plant species, has been shown to exhibit neuroprotective, anti-oxidative, anti-inflammatory, and cognitive-enhancing effects. However, the effects of α-asarone on spinal cord injury (SCI) have not yet been elucidated. The present study investigated the effects of α-asarone on the mRNA of pro-inflammatory cytokines, macrophage polarization toward an anti-inflammatory M2 phenotype, and angiogenesis in rats with compressive SCI. α-Asarone was orally administered (10 mg/kg) once per day for 14 days following moderate static compression SCI. Compared to controls, α-asarone treatment significantly improved locomotor score, prevented neuroinflammation, and facilitated angiogenesis in the spinal cord at 14 days after SCI. Furthermore, α-asarone significantly reduced the TNF-α, IL-1β, IL-6, monocyte chemoattractant protein 1 (MCP-1), macrophage inflammatory protein 2 (MIP-2), and inducible nitric oxide synthase (iNOS) levels but increased the IL-4, IL-10, and arginase 1 levels at 24 h after SCI. At 7 and 14 days after SCI, immunohistochemistry showed reduced reactive gliosis and neuroinflammation and an increased expression of M2 macrophage markers and angiogenesis. The results suggest that the inhibition of pro-inflammatory cytokines, macrophage polarization toward an anti-inflammatory M2 phenotype, and angiogenesis by α-asarone may be some of the mechanisms underlying the α-asarone-mediated neuroprotective effects on an injured spinal cord.

## Introduction

Spinal cord injury (SCI) is a two-step process that includes a primary mechanical injury and a subsequent secondary injury mediated by multiple injury processes, including inflammation, apoptosis, free radical-induced cell death, and glutamate excitotoxicity (Bharne et al., 2013; Cheng et al., 2014; Losey et al., 2014). After SCI, inflammatory responses are a major component of secondary injury. The blood–spinal cord barrier (BSCB) is disrupted, and the injury site is rapidly infiltrated by blood-borne neutrophils. Thus, inflammatory responses following SCI are initiated by peripherally derived immune cells and activated glial cells that proliferate or migrate into the lesion site. This influx of inflammatory cells induces the apoptosis of neurons and oligodendrocytes and the formation of glial scars, and finally results in the reduction of neuronal function (Zhou et al., 2014; Shultz and Zhong, 2017). This inflammatory process not only deteriorates macrophage responses and changes the polarization state of macrophages, which is thought to depend in large part on cytokines and other immune cells in SCI neurons, but it also retards the recovery process (Hu et al., 2015). Macrophages play an essential role in the non-specific immune response that protects the body from pathogens after SCI (Kroner et al., 2014). After such an injury, monocytes migrate to the damaged region and differentiate into M1 macrophages (David and Kroner, 2011). M1 macrophages stimulate phagocytosis through the release of inflammatory cytokines, such as tumor necrosis factor α (TNF-α), interleukin (IL)-1β, and IL-6, and they digest myelin, red blood cells, and dead neuronal cells. After this process, M1 macrophages can transform into M2 macrophages, which release factors such as IL-4 and IL-10. These cytokines, in turn, induce M2 polarization through autocrine and paracrine actions (Zhou et al., 2014). M2 macrophages decrease the phagocytic activity of macrophages and release anti-inflammatory cytokines, growth factors, and angiogenic factors, thereby reducing inflammatory immune reactions and assisting in the regeneration of neural tissues (Hu et al., 2015). It has been hypothesized that increasing the numbers of M2 macrophages may significantly improve the functional regenerative activity of the spinal cord in case of injury. Some studies have shown that regulating the polarization of M2 macrophages can prevent secondary damage after SCI and assist in the regeneration of the spinal cord (Kigerl et al., 2009; Wang et al., 2015). Thus, reducing the levels of pro-inflammatory cytokines is believed to be a potential therapy for SCI (Shultz and Zhong, 2017). Methylprednisolone, which is a corticosteroid medicine, is the only pharmaceutical drug approved by the Food and Drug Administration that can alleviate inflammation. Although some research has underlined the effects of this compound on reducing inflammation, methylprednisolone has many side effects, such as sepsis, gastrointestinal bleeding, and pneumonia (Harrop, 2014). These serious side effects have limited the use of methylprednisolone in clinical trials. Given the conflicting evidence from multiple clinical trials (Hall and Springer, 2004; Evaniew and Dvorak, 2016), many centers have abandoned the use of methylprednisolone in the acute treatment of SCI. Thus, developing a compound with potent therapeutic efficacy and fewer side effects has been an unmet clinical need for SCI treatment.

α-Asarone is a bioactive component derived from Acorus plant species and an Eastern traditional herb that is well known for its effectiveness in central nervous system (CNS) disorders and cognitive functions (Kim et al., 2015). This compound is known for its neuroprotective function through the blockade of the N-methyl-D-aspartate (NMDA) receptor and by inhibiting the nitric oxide (NO) overproduction in various brain regions (Shin et al., 2014). α-Asarone also inhibits neuroinflammation by suppressing pro-inflammatory cytokines such as TNF-α, IL-6, and IL-1β (Kumar et al., 2012; Shin et al., 2014; Kim et al., 2015). Additionally, it has the potential to effectively reach the CNS, as it is known to cross the blood–brain barrier (Lu et al., 2015), and to induce angiogenesis, which is essential in neural regeneration (Park et al., 2015). These previous studies have demonstrated that α-asarone can enhance functional recovery after SCI. Thus, we investigated the effect of α-asarone on pro-inflammatory cytokine mRNA, macrophage polarization toward an anti-inflammatory M2 phenotype, and angiogenesis in rats with compressive SCI.

## Materials and Methods

### Animals

Young adult female Sprague-Dawley rats (200–220 g body weight, *n* = 54) were used for this study. This study and the research protocols were carried out in accordance with and approved by the Institutional Animal Care and Use Committee (IACUC) of CHA University (IACUC170037). All animals were purchased from Orient Bio Inc. (Seongnam, South Korea). Animals were housed in plastic cages at constant temperature of 24 ± 3°C with a relative humidity of 60 ± 5% under a 12-h light–dark cycle. The animals were allowed free access to food and water before the experiment.

### Surgical Model of Moderate Static Compression SCI

**FIGURE 1:**
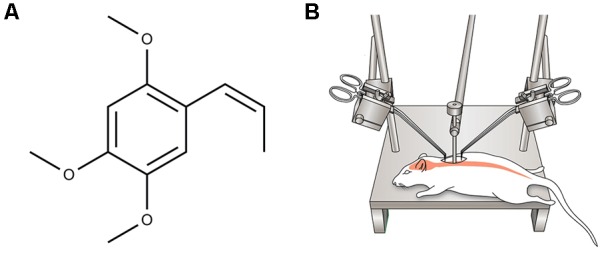
**(A)** Chemical structure of α-asarone. **(B)** A schematic diagram showing moderate static compression spinal cord injury (SCI) rat model induced by imposing 35 g of static weight on the dorsal surface of the T10 spinal cord for 5 min.

All animals were anesthetized with ketamine (100 mg/kg i.p.) and xylazine (10 mg/kg i.p.) before operation. Dorsal laminectomy at thoracic vertebra 10 (T10) was performed to expose the spinal cord. The rat body was suspended with an INBO laboratory spine stabilization frame (fabricated at the CHA University, Seongnam, South Korea), with a 1-cm clearance between the ventral disc of the rat and the frame base platform. After flattening of the spine, a 35 g stainless steel impounder was gently placed on the dorsal surface of the spinal cord through a micromanipulator. A moderate compressive damage is delivered to the bar to deliver a 5-min waiting load (Kumar et al., 2017; **Figure 1B**). Following the compression injury, muscle and skin incisions were closed using appropriate monofilament sutures (Silk 4-0). Animals were then injected subcutaneously with 5 mL of 0.9% sterile saline and placed on a heating pad to maintain body temperature until they were conscious. The urinary bladder was gently pressed twice a day manually until a normal bladder reflex was established.

### α-Asarone Preparation and Treatment

α-Asarone (trans-1-Propenyl-2,4,5-trimethoxybenzene, **Figure 1A**) was purchased from Sigma-Aldrich (St. Louis, MO, United States). Thirty minutes after SCI, α-asarone (10 mg/kg) dissolved in 0.5% carboxymethylcellulose (CMC) solution and 1% Tween80 [Polyoxyethylene (20) sorbitan monooleate] were orally administered once a day for 14 days. The 54 rats were divided into three groups: the sham (*n* = 6), control (*n* = 24, injury only), and α-asarone treatment groups (*n* = 24). The sham group (laminectomy only) was allowed free access to food and water without any treatment. The control group received vehicle (0.5% CMC solution containing 1% Tween80) for 14 days.

### Animal Locomotion Test

To test hind limb locomotor function, an open-field locomotion test (*n* = 10/group) based on the Basso, Beattie, Bresnahan (BBB) scale (Basso et al., 1995), was completed at 1, 2, 3, 4, 5, 6, 7, and 14 days after SCI. During the evaluation, animals were allowed to walk freely on the open-field surface for 3 min while being observed by two blinded scorers.

### Quantitative Real-Time Polymerase Chain Reaction (RT-PCR)

The rats were sacrificed by carbon monoxide intoxication, and the injured spinal cord was rapidly transferred on ice. Total RNA from 1 cm of the spinal cord segment with the lesion site approximately at the middle were homogenized using T 25 digital homogenizer (IKA, Seoul, South Korea) in TRIzol reagent (Invitrogen, CA, United States) according to the manufacturer’s instructions for RNA extraction. Complementary DNA was synthesized from 1 μg of total RNA using a cDNA using synthesis kit (TAKARA, Shiga, Japan). Quantitative real-time PCR was performed using an SYBR Green Master Mix, and the detection of mRNA was analyzed using an ABI StepOne Real-time PCR System (Applied Biosystems, CA, United States). The PCR protocol consisted of 40 cycles of denaturation at 95°C for 15 s, followed by 60°C for 30 s to allow for extension and amplification of the target sequence. Pro-inflammatory cytokines (TNF-α, IL-1β, and IL-6), anti-inflammatory cytokines (IL-4, IL-10), chemokine [monocyte chemoattractant protein 1 (MCP-1), macrophage inflammatory protein 2 (MIP-2)], and M1 and M2 markers [inducible NO synthase (iNOS) and arginase 1 (Arg 1), respectively] were measured by quantitative RT-PCR 24 h after SCI. The primers were procured from Bioneer (Daejeon, South Korea). The primer sequences used in this study are shown in **Table 1**. The expression levels of target genes were normalized to GAPDH and compared with the control set-up. Data were analyzed using the ΔΔCT method (Schmittgen and Livak, 2008). These experiments were repeated three times.

**Table 1:** Nucleotide sequences of primers used in real-time qRT-PCR.

Gene	Forward (5′–3′)	Reverse (5′–3′)
TNF-α	AGCAAACCACCAAGTGGAGGA	GCTGGCACCACTAGTTGGTTGT
IL-1β	AGTTGACGGACCCCAAAAG	AGCTGGATGCTCTCATCAGG
IL-6	GCTACCAAA CTGGATATAAT CAGGA	CCAGGTAGCTATGGTACTCCAGAA
IL-4	GGTCTCAGCCCCCACCTTGC	CCGTGGTGTTCCTTGTTGCCGT
IL-10	CAGAGCCACATGCTCCTAGA	TGTCCAGCTGGTCCTTTGTT
MCP-1	TCACGCTTCTGGGCCTGTTG	CAGCCGACTCATTGGGATCATC
MIP-2	GGCACAATCGGTACGATCCAG	ACCCTGCCAAGGTTGACTTC
Arg 1	TTGATGTTGATGGACTGGAC	TCTCTGGCTTATGATTACCTTC
iNOS	GAGTGAGGAGCAGGTTGAGG	CCAAGGTGTTGCCCTTTTT
GAPDH	AGGTCATCCCAGAGCTGAACG	CACCCTGTTGCTGTAGCCGTAT

*GAPDH, glyceraldehyde-3-phosphate dehydrogenase; IL, interleukin; iNOS, inducible nitric oxide synthase; MCP-1, macrophage chemoattractant protein 1; MIP-2, macrophage inflammatory protein 2; TNF-α, tumor necrosis factor α.*

### Immunohistochemistry

After the behavior test, the rats were deeply anesthetized and perfused transcardially with 0.05 M phosphate-buffered saline (PBS) containing 4% paraformaldehyde. The spinal cord was removed and post-fixed in the same perfusion solution overnight at 4°C. The spinal cord tissues were embedded in paraffin, sectioned at a 5-μm thickness, deparaffinized, and then treated with 0.3% hydrogen peroxide in methyl alcohol for 10 min to block endogenous peroxidase activity. After three washes with PBS, the blocking (GBI, WA, United States) reagent was added, and staining was performed with antibodies against glial fibrillary acidic protein (GFAP, 1:200; Sigma, St. Louis, MO, United States), CD68 (1:200; Abcam, Cambridgeshire, United Kingdom), arginase 1 (Arg 1, 1:120; Abcam), iNOS (1:100; Abcam), angiopoietin 1 (Ang 1, 1:200; Abcam) and vascular endothelial growth factor receptor 1(VEGFR1, 1:250; Abcam). Sections of the spinal cord samples were incubated for 1 h with an appropriate secondary antibody conjugated to Alexa 488, Alexa 568, or Alexa 647 (1:200; Invitrogen) fluorophores. DAPI (1:10000; Invitrogen, CA, United States) was used as a counterstain to visualize nuclei. Confocal images of spinal cord longitudinal sections were obtained with a Zeiss LSM 880 with Airyscan confocal system in a sequential scanning mode. Fluorescence intensity was analyzed semi-quantitatively by image analysis ZEZ 2012 software (Zeiss).

### Hematoxylin-Eosin (HE) Staining

The 10-μm-thick sections were rinsed in distilled water and submerged in hematoxylin solution for 3 min, followed by a quick de-staining in 0.5% acid methyl alcohol for 10 s. Then, the tissues were washed with tap water for 10 min and stained with eosin for 2 min. Finally, the sections were dehydrated through an increasing series of ethanol concentration (70, 80, 95, and 100% alcohol) for 2 min each.

### *In Vitro* Cell Culture

To investigate the effects of α-asarone on macrophage polarization, we used the immortalized murine macrophage cell line RAW 264.7 *in vitro*. Cells were grown in complete RPMI 1640 medium (HyClone, Logan, United States) containing penicillin (100 U/ml), streptomycin (100 U/ml), and 10% FBS (HyClone), maintained at 37°C and 5% CO2. Briefly, 5 × 10^3^ cells were seeded into 12-well cell culture plates (Falcon Becton Dickinson, Lincoln Park, NJ, United State) and cultured for 24 h. Then, cells were stimulated with lipopolysaccharide (LPS, Sigma-Aldrich) for 24 h. Subsequently, α-asarone (0, 10, 50, 250 μM) was added, and cells were incubated for another 24 h. Then, cells were placed on slides and were prepared for immunocytochemical staining. To detect M1 macrophage markers, we used a rabbit antibody against iNOS (1:200; Abcam). To detect M2 markers, we used an antibody against Arg 1 (1:200; Abcam). A CD68 (1:500; Abcam) antibody was used as a pan macrophage marker. To detect cytokines, we used antibodies against monoclonal IL-6 (1:200; Abcam) and IL-10 (1:200; Abcam). All samples were incubated for 1 h with the appropriate secondary antibody conjugated to Alexa 488, Alexa 568, or Alexa 647 (1:200; Invitrogen) fluorophores. DAPI (1:10000; Invitrogen) was used as a counterstain to visualize nuclei. Cells were observed by using confocal microscopy (Zeiss LSM 880 with Airyscan). Five different fields of each slide were acquired, and pictures of each group were collected from triple-cultured cells. The fluorescence intensity of IL-6 and IL-10 was analyzed semi-quantitatively using the image analysis ZEZ 2012 software (Zeiss).

### Statistical Analyses

All data were analyzed using Graph Pad Prism ver. 5.01 (Graph Pad, Inc., La Jolla, CA, United States). All data are expressed as the mean ± standard error mean of at least three independent experiments performed in triplicate. The statistical analysis was performed with one-way factorial analysis of variance (ANOVA) with *post hoc* Tukey test, one-tailed Student’s *t*-test, and Wilcoxon rank sum test. *P*-values < 0.05 were considered statistically significant.

## Results

### α-Asarone Improves Functional Recovery After SCI

**FIGURE 2:**
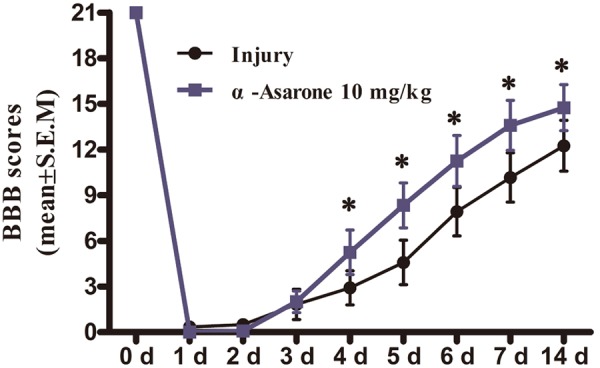
The effect of α-asarone on locomotor assessment. Basso, Beattie, and Bresnahan (BBB) scores for rats in the SCI (*n* = 6) and α-asarone-treated (*n* = 6) groups at different time points (1, 2, 3, 4, 5, 6, 7, and 14 days) post-surgery. ^∗^*P* < 0.05. Data are represented as the mean ± standard error of the mean and analyzed using Wilcoxon rank sum tests.

Compared to the injury group, α-asarone treatment showed a significant increase in functional recovery after SCI (**Figure 2**). The results indicate that α-asarone was effective in ameliorating functional deficits in rats with compressive SCI.

### α-Asarone Promotes M2 Macrophage Polarization in LPS-Treated RAW 264.7 Macrophage Cells

**FIGURE 3:**
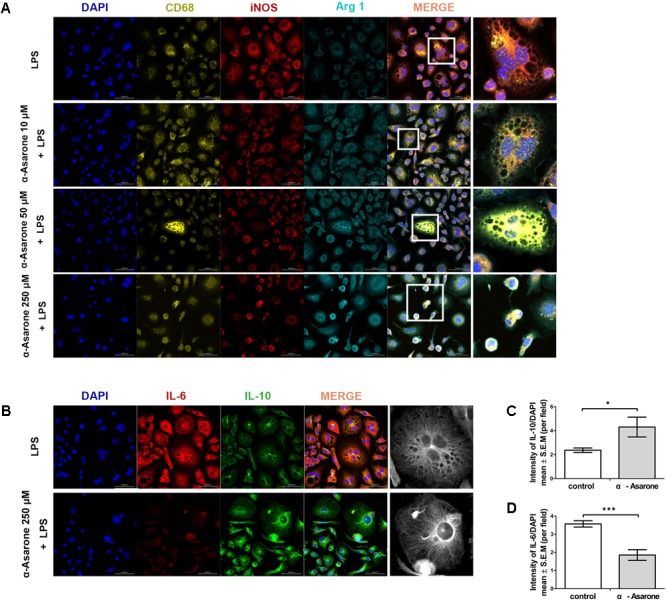
α-asarone promotes M2 macrophage polarization *in vitro*. **(A)** Immunolabeling for CD68 (yellow, macrophage marker), inducible nitric oxide synthase (iNOS; red, M1 marker), and Arginase 1(Arg 1; turquoise, M2 marker), DAPI (4′,6-diamidino-2-phenylindole) is used for nuclear counterstaining. Scale bars, 50 μm. **(B)** Immunolabeling for interleukin-6 (IL-6, red) and interleukin-10 (IL-10, green). Scale bars, 50 μm. **(C)** Quantitative analysis of immunohistochemistry for IL-6. ^∗^*P* < 0.05, one-tailed Student’s t-test (n = 5). **(D)** Quantitative analysis of immunohistochemistry for IL-10. ^∗∗∗^*P* < 0.001, one-tailed Student’s *t*-test (*n* = 5).

The M1 macrophage phenotype was first induced by *in vitro* LPS stimulation (1 μg/ml) for 24 h. It was possible to observe morphological changes in RAW 264.7 cells after inducing inflammation using LPS, but the morphological changes were different between cells treated with α-asarone and the vehicle control. As shown in **Figure 3A**, the vehicle-treated cells exhibited elevated levels of iNOS, an M1 marker, and low levels of Arg 1, an M2 marker. The levels of iNOS, as assessed by the fluorescence intensity, were gradually decreased in cells treated with 10 μM, 50 μM, or 250 μM of α-asarone compared to control-treated cells. Arg 1 fluorescence intensity was also increased in cells treated with 250 μM of α-asarone compared to the vehicle-treated cells. In addition, the vehicle-treated cells had high levels of IL-6, a pro-inflammatory cytokine, after LPS stimulation. α-Asarone attenuated the fluorescence intensity of IL-6 (**Figures 3B,C**) but increased that of IL-10, an anti-inflammatory cytokine (**Figures 3B,D**). These results indicate that α-asarone induced macrophage polarization toward an anti-inflammatory M2 phenotype in the RAW 264.7 macrophage cells upon treatment with LPS.

### α-Asarone Inhibits Pro-inflammatory Cytokines and Inflammatory Mediators After SCI

**FIGURE 4:**
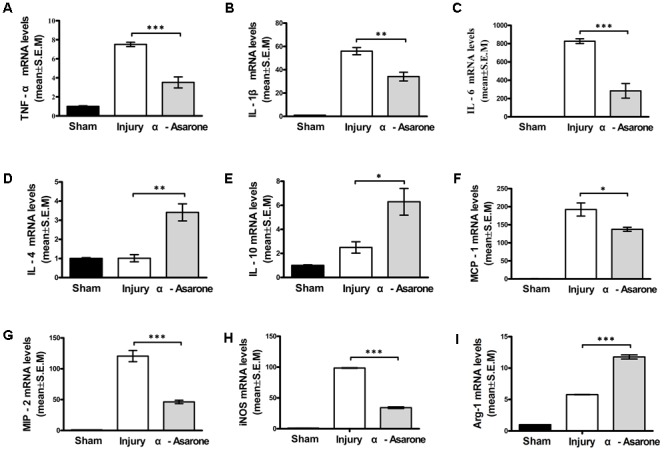
Effects of α-asarone on mRNA expression levels of cytokines and chemokines in injured spinal cord as measured using real-time RT-PCR. **(A)** TNF-α, **(B)** IL-1β, **(C)** IL-6, **(D)** IL-4, **(E)** IL-10, **(F)** monocyte chemotactic protein (MCP)-1, **(G)** macrophage inflammatory protein (MIP)-2, **(H)** inducible nitric oxide synthase, and **(I)** Arg 1 mRNA levels. The relative expression levels of target mRNAs were calculated using the ΔΔ Ct method and expressed relative to the value in the sham group (designated as 1). Data represent the mean ± standard error of the mean of three independent experiments (*n* = 3). ^∗^*P* < 0.05, ^∗∗^*P* < 0.01, and ^∗∗∗^*P* < 0.001. Statistical comparisons were performed using one-way analyses of variance followed by Tukey tests.

One day after SCI, the mRNA expression levels of pro-inflammatory cytokines TNF-α, IL-1β, and IL-6 were significantly lower in the α-asarone-treated group than they were in the injury group (**Figures 4A–C**). In contrast, the expression levels of the anti-inflammatory cytokines IL-4 and IL-10 were upregulated in the α-asarone-treated group compared to the injury group (**Figures 4D,E**). Moreover, the expression levels of inflammatory chemokines, MCP-1 and MIP-2 were significantly lower in the α-asarone-treated group than they were in the injury group (**Figures 4F,G**). In addition, the levels of the M1 marker iNOS were significantly decreased in the α-asarone-treated group compared to the injury group (**Figure 4H**). The levels of the M2 marker, Arg 1 were increased in the α-asarone-treated group compared to the injury group (**Figure 4I**). These results suggest that α-asarone may reduce the expression of pro-inflammatory cytokines in the injured spinal cord, induce an anti-inflammatory effect, and cause macrophage polarization.

### α-Asarone Attenuates Secondary Damage and Prevents Glial Scar Formation in Rats With SCI

**FIGURE 5:**
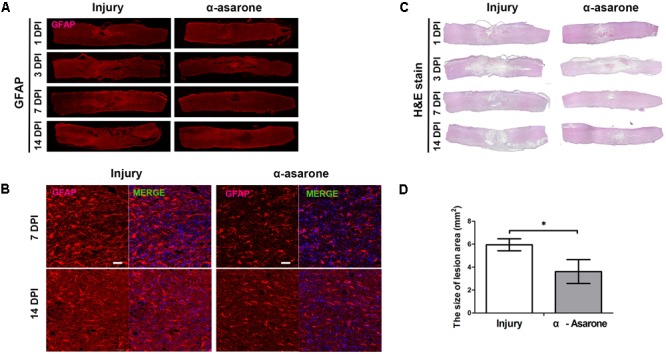
Histopathological and immunohistochemical analyses of the spinal cord. **(A)** Immunolabeling for GFAP (red, glial fibrillary acidic protein) of longitudinal sections of spinal cords from rats subjected to spinal cord injury (Injury) or α-asarone treatment following injury (α-asarone). DPI, days post injury. Spinal cord length size, 1 cm. **(B)** Histological findings were based on hematoxylin-eosin staining of longitudinal sections of spinal cords from rats in the Injury or α-asarone group, respectively. **(C)** Immunolabeling for GFAP (red) and DAPI (4′,6-diamidino-2-phenylindole) nuclear staining. Scale bars, 50 μm. **(D)** The graph shows the mean area of scar in mm^2^ ± SD after 14 days following SCI ^∗^*P* < 0.05, one-tailed Student’s *t*-test (*n* = 6).

The expression of the GFAP protein, which is a marker of reactive gliosis and neuroinflammation, in the injured spinal cord tissue was measured at 1, 3, 7, and 14 days after SCI using immunohistochemistry. At each time point, α-asarone treatment showed reduced immunoreactivity for GFAP at the lesion site, compared to the vehicle-treated group (**Figure 5A**). HE staining (**Figure 5B**) of the longitudinal spinal cord revealed hemorrhage and neuronal loss in the epicenter in rats subjected to SCI 1 dpi in both groups. The hemorrhage gradually disappeared 3 days after injury. α-asarone treatment induced a significant decrease in the lesion area at the epicenter compared to the injury group (**Figure 5B**). The size of the lesion area and the tissue loss were reduced in the α-asarone-treated group compared to the injury group 7 days after injury (**Figure 5B**). At 7 and 14 days after injury, immunohistochemical staining of the transverse spinal cord demonstrated that immunoreactivities for GFAP were significantly decreased in α-asarone-treated group (**Figure 5C**). Furthermore, the mean area of the lesion cavity was 5.94 ± 1.28 mm^2^ in the injury group 14 days after injury. However, the α-asarone-treated group revealed a smaller lesion cavity area of 3.60 ± 2.55 mm^2^ (one-tailed Student’s *t*-test, *P* < 0.05; **Figure 5D**). These results demonstrate that α-asarone can reduce the lesion cavity with tissue protection.

### α-Asarone Induces Macrophage Polarization Toward an Anti-inflammatory M2 Phenotype in Rats With SCI

**FIGURE 6:**
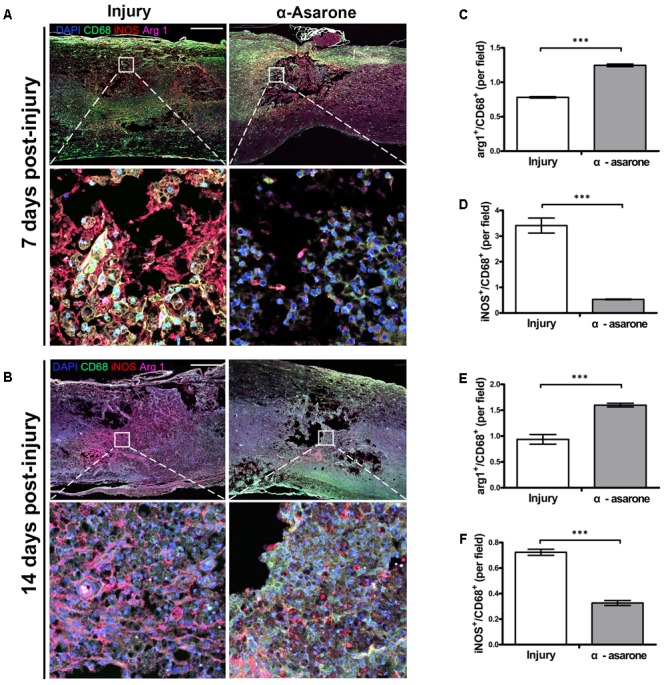
α-asarone triggered M2 macrophage phenotype *in vivo*. **(A,B)** Immunolabeling for CD68 (green, microglia, and macrophage maker), iNOS (red, M1 marker), and Arg 1 (purple, M2 marker). DAPI is used for nuclear counterstaining at the lesion site. Scale bars, 5,000 μm and 50 μm. **(C–F)** Semi-quantitative analysis of immunohistochemistry for Arg 1 and iNOS. ^∗∗∗^*P* < 0.001, one-tailed Student’s *t*-test (*n* = 5).

iNOS was selected as a marker for macrophages with an M1 phenotype and Arg 1 as a marker for macrophages with an M2 phenotype. The expression of iNOS and Arg 1 was semi-quantitatively evaluated in an injured spinal cord. Representative stainings of infiltrating iNOS or Arg 1 macrophages are shown in **Figure 6**. At 7 (**Figure 6A**) and 14 days (**Figure 6B**) after SCI, Arg 1 expression was significantly higher in the α-asarone-treated group than in the injury group. In addition, the M1 marker iNOS had significantly lower levels in the α-asarone-treated group than in the injury group (**Figure 6**). These results reveal that induces macrophage polarization toward an anti-inflammatory M2 phenotype after SCI.

### α-Asarone Promotes Angiogenesis in Rats With SCI

**FIGURE 7:**
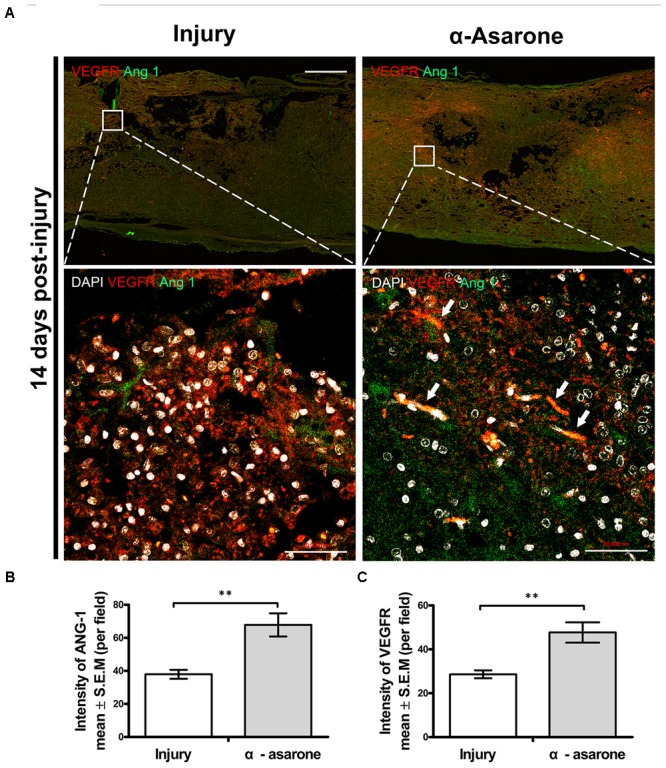
α-asarone promoted angiogenesis *in vivo*. **(A)** Immunolabeling for vascular endothelial growth factor receptor (VEGFR; red, angiogenesis marker), Ang 1 (green, angiogenesis marker). DAPI is used for nuclear counterstaining at the site of the lesion. Scale bars, 5,000 μm. **(B,C)** Quantitative analysis of immunohistochemistry for VEGFR, Ang 1. ^∗∗^*P* < 0.01, one-tailed Student’s *t*-test (*n* = 5).

We performed immunohistochemical analysis to examine changes in the expression levels of angiogenic markers (VEGFR, Ang 1) in the spinal cord epicenter. The expression of VEGFR and Ang 1was semi-quantitatively evaluated in an injured spinal cord. Fourteen days after SCI, immunohistochemical staining showed increased immunoreactivity for VEGFR and Ang 1 in the α-asarone-treated group (**Figure 7**). In the larger magnification picture, blood vessel morphology was observed in the α-asarone-treated group (**Figure 7A**). In addition, semi-quantitative evaluation staining showed an increased intensity of VEGFR and Ang 1 in α-asarone-treated group than in the injury group (**Figures 7B,C**).

## Discussion

Accumulating evidence indicates that α-asarone has neuroprotective effects that can be used as a treatment for SCI (Shin et al., 2014). The present study showed that α-asarone enhanced functional recovery in rats with compressive SCI. This *in vivo* study showed that α-asarone reduced the over-production of pro-inflammatory cytokines (TNF- α, IL-1β, IL-6), chemokines such as MCP-1 and MIP-2 and reactive gliosis and neuroinflammation. α-Asarone also increased the anti-inflammatory cytokines (IL-4 and IL-10) and the expression of angiogenic factors. In addition, α-asarone increased the expression of a M2 macrophage marker and decreased the expression of a M2 macrophage marker, which suggests macrophage polarization toward an anti-inflammatory M2 phenotype after α-asarone treatment.

Within the area of the SCI, macrophages contribute to secondary pathological and inflammatory responses in part through the release of cytokines (Detloff et al., 2008; Zhang et al., 2012). However, alternatively activated macrophages (M2) have positive effects on repair, growth, and regenerative processes following SCI (Gensel and Zhang, 2015). A recent study reported that some M2 macrophages migrated to the spinal cord lesion via the choroid plexus and moved along the central canal down to the lesion (Shechter et al., 2013). Blocking the entry of these cells along this pathway hindered locomotor recovery, which suggests that M2 macrophages contribute to locomotor recovery after SCI (Shechter et al., 2013). For this reason, the macrophage response following injury needs to be regulated and shifted toward protection and repair without completely blocking macrophage actions. Controlling the polarization of M2 macrophages would facilitate the development of new therapies for patients with SCI and potentially allow improved responses to the currently available therapies.

A strategy for ameliorating secondary injury may be reducing the levels of anti-inflammatory cytokines and increasing the levels of anti-inflammatory cytokines to promote the conversion of M1 macrophages to M2 macrophages (Hayakawa et al., 2014; Martinez and Gordon, 2014; Makita et al., 2015). As indicated in previous reports, α-asarone can reduce oxidative stress and has anti-inflammatory effects (Kumar et al., 2012; Shin et al., 2014; Kim et al., 2015). The findings from these studies are consistent with those of our *in vitro* experiments. α-asarone significantly reduced the IL-6 levels and significantly increased the IL-10 levels after the induction of M1 macrophages. Furthermore, we confirmed that the induced M1 macrophages were inactivated by the anti-inflammatory effects of α-asarone and were converted into M2 macrophages.

In the first few days after SCI, the levels of pro-inflammatory cytokines, such as TNF-α, IL-6, and IL-1β, were all increased, mostly due to macrophage actions (Donnelly and Popovich, 2008; Zhang et al., 2012). The levels of inflammatory chemokines MCP-1 and MIP-2 were also increased (Cheng et al., 2014; Zhang et al., 2015). These phenomena suggest that the increased inflammatory response after SCI leads to secondary injury, which involves apoptosis, neuronal necrosis, and the activation of macrophage cells, and interferes with the recovery of function (Donnelly and Popovich, 2008; Cheng et al., 2014). Our *in vivo* results support the expectation that α-asarone has anti-inflammatory effects. We measured the levels of pro-inflammation markers that can affect secondary injury in a rat model of SCI. In particular, on the 1st day after SCI, the mRNA levels of the inflammatory cytokines IL-6, TNF-α, and IL-1β and those of the chemokines MCP-1 and MIP-2 were significantly increased in the injury group. In contrast, the IL-6, TNF-α, IL-1β, MCP-1 and MIP-2 levels were significantly decreased in α-asarone-treated animals. Furthermore, α-asarone treatment after SCI was associated with a significant increase in the mRNA levels of the anti-inflammatory cytokines IL-4 and IL-10 compared to control treatment. These anti-inflammatory effects of α-asarone have the potential to reduce secondary injury.

During the secondary injury phase following SCI, M1 macrophages invade the lesion and release inflammatory cytokines (Silver and Miller, 2004). These M1 macrophages then contribute to the formation of axonal growth-inhibitory glial scars by reactive astrocytes, which are important impediments to locomotor recovery (Silver and Miller, 2004; Shechter et al., 2013). In addition, the infiltration of M1 macrophages into glial scars contributes to axonal dieback (Zhou et al., 2014). Thus, the conversion of M1 macrophages into M2 macrophages can inhibit glial scar formation and axonal dieback (Kong and Gao, 2017). Our results also indicate that both glial scar formation and the size of the lesion area were decreased in the spinal cord in rats treated with α-asarone compared to those in the injury group 7 days after SCI.

Additionally, M2 macrophages release angiogenic factors such as Ang 1 and VEGFR. These factors can promote angiogenesis, which may improve tissue perfusion during the repair stage after spinal cord trauma. Moreover, angiogenesis is closely associated with neurogenesis. From this perspective, M2 macrophages are believed to participate actively in neurogenesis (Ritz et al., 2010; Hayakawa et al., 2014; Jetten et al., 2014), which is essential for functional recovery after SCI. A previous study has shown that α-asarone can stimulate angiogenesis in an *ex vivo* aorta model in a way similar to the wound healing process (Park et al., 2015). In accordance with this finding, our results showed that the application of α-asarone significantly upregulated the expression levels of Ang 1 and VEGFR around the lesion area. As a result, more blood vessels were observed in the α-asarone-treated group. The number of M2 macrophages and the levels of angiogenesis markers both increased around the lesion site in the α-asarone-treated group. Our results may indicate that α-asarone may facilitate angiogenesis by skewing the polarization of macrophages toward the M2 subtype. Taking these results together, the improvement of hind limb locomotor function after SCI may be attributed to the anti-inflammatory effects and subsequent pro-angiogenesis properties of α-asarone.

α-Asarone is usually administered orally via capsules and tablets in clinical settings. In this study, we adopted the previously established dose of α-asarone (10 mg/kg daily) for oral administration. The effectiveness of a compound can vary by delivery method; for example, recent studies have shown that the nasal delivery of α-asarone is more effective than oral administration (Lu et al., 2015). Therefore, future research could be carried out to assess the therapeutic potential of this drug when delivered via different routes that may be more convenient for patient application. Moreover, given the concern of potential toxicity in hepatocytes (López et al., 1993; Karwicka et al., 2008), therapeutic regimens should be carefully tailored, leveraging benefits out of the possible side effects.

## Conclusion

This work suggests that the application of α-asarone can promote motor function recovery of rats following SCI, presumably due to its multiple actions on attenuating inflammation, such as skewing the polarization of macrophages toward M2 subtype and facilitating tissue repair. Our findings show the effectiveness of α-asarone in managing SCI in a clinical-relevant animal model. The putative therapeutic effects together with the easy route of administration render α-asarone a potential candidate for use in treating SCI.

## Author Contributions

M-JJ and IH conceived and directed the project. M-JJ and HK designed the experiments. M-JJ, HJ, W-KK, HC, JK, SH, SP, SS, AB, and S-HL carried out the experiments. SP, SS, AB, K-TK, and S-HL conducted the data analysis and interpreted the results. M-JJ, IH, and XZ wrote and edited the paper.

## Conflict of Interest Statement

The authors declare that the research was conducted in the absence of any commercial or financial relationships that could be construed as a potential conflict of interest.
