# Corrigendum: Oral Administration of α-Asarone Promotes Functional Recovery in Rats With Spinal Cord Injury

**DOI:** 10.3389/fphar.2018.00949

**Published:** 2018-08-30

**Authors:** Min-Jae Jo, Hemant Kumar, Hari P. Joshi, Hyemin Choi, Wan-Kyu Ko, J. M. Kim, Sean S. S. Hwang, Song Y. Park, Seil Sohn, Alvin B. Bello, Kyoung-Tae Kim, Soo-Hong Lee, Xiang Zeng, Inbo Han

**Affiliations:** ^1^Department of Neurosurgery, CHA Bundang Medical Center, CHA University, Seongnam-si, South Korea; ^2^Department of Biomedical Science, CHA University, Seongnam-si, South Korea; ^3^Department of Neurosurgery, School of Medicine, Kyungpook National University, Kyungpook National University Hospital, Daegu, South Korea; ^4^Department of Histology and Embryology, Zhongshan School of Medicine, Sun Yat-sen University, Guangzhou, China

**Keywords:** spinal cord injury, α-asarone, neuroprotection, anti-inflammation, M2 polarization

In the published article, there was an error in affiliation 3. The School of Medicine and Kyungpook National University were not mentioned. The authors apologize for this error and state that this does not change the scientific conclusions of the article in any way.

The original article has been updated.

## Conflict of interest statement

The authors declare that the research was conducted in the absence of any commercial or financial relationships that could be construed as a potential conflict of interest.

